# Proteomic Profiling of Myofiber Repair Annexins and Their Role in Duchenne Muscular Dystrophy

**DOI:** 10.1002/pmic.70073

**Published:** 2025-11-11

**Authors:** Paul Dowling, Dounia Bouragba, Elisa Negroni, Capucine Trollet, Margit Zweyer, Dieter Swandulla, Kay Ohlendieck

**Affiliations:** ^1^ Department of Biology Maynooth University National University of Ireland Maynooth Co. Kildare Ireland; ^2^ Kathleen Lonsdale Institute for Human Health Research Maynooth University Maynooth Co. Kildare Ireland; ^3^ Center for Research in Myology U974 Sorbonne Université, INSERM Paris France; ^4^ German Centre for Neurodegenerative Diseases Bonn Germany; ^5^ Institute of Physiology Faculty of Medicine University of Bonn Bonn Germany

**Keywords:** annexin, caveolin, dysferlin, dystrophin, myoferlin

## Abstract

Myofiber regeneration and membrane repair play crucial roles in maintaining the continuous physiological functioning of the neuromuscular system. A swift and efficient repair mechanism enables the rapid restoration of sarcolemmal integrity following cellular impairment in damaged skeletal muscles. Members of the annexin family of proteins, which are characterized by the peripheral binding to acidic phospholipid membranes, are intrinsically involved in this myofiber repair process. The biochemical and proteomic profiling of dystrophinopathy, a severe and highly progressive neuromuscular disorder of early childhood, is outlined in this article with special focus on skeletal muscle‐associated annexins and their role in membrane repair, myofiber regeneration and the cellular pathogenesis of Duchenne muscular dystrophy. Findings from comparative mass spectrometry‐based surveys are described, and dystrophinopathy‐related alterations in annexin expression patterns are discussed regarding the establishment of improved biomarker signatures of skeletal muscle wasting disorders. Mass spectrometry‐based proteomic profiling is highly suitable for the systematic study of complex pathobiochemical alterations and inherent adaptations in dystrophinopathy. Disease‐specific changes in annexins and related proteins of the membrane repair machinery can now be used to improve diagnosis, evaluation of disease severity, prognosis and therapeutic monitoring, and identify novel therapeutic targets to treat X‐linked muscular dystrophy.

## 1 Introduction

The skeletal musculature, which is characterized by longitudinal arrays of multi‐nucleated myofibers that represent the most abundant tissue type in the human body [1], is under continuous physical strain [2]. High levels of cellular damage can impair the physiological fine‐tuning of muscle functions and disrupt the integrity of the excitation‐contraction‐relaxation cycle. Therefore, cellular responses to muscle damage are crucial for maintaining the essential properties of the voluntary contractile system [3], including metabolic flexibility, tissue elasticity, myofiber contractility and physiological plasticity [4]. These essential biological features are responsible for body‐wide functionality related to posture, directional movements, protection, breathing, communication, bioenergetic integration and heat homeostasis [5].

Skeletal muscular damage can be triggered by traumatic injury, crush syndrome, and strenuous physical exercise, as well as primary or secondary muscle diseases in association with acquired, autoimmune or genetic factors. Severe muscular dysfunction is seen in muscular atrophies, muscular dystrophies, pharmacogenetic myopathies, channelopathies and motor neuron diseases, and is also associated with certain systemic disorders, such as cancer cachexia, diabetes mellitus, sepsis and the age‐related frailty syndrome [3, 6, 7, 8]. Within the large group of muscular dystrophies [9], the most frequently inherited neuromuscular disorder of early childhood is Duchenne muscular dystrophy (DMD) [10]. DMD is characterized by primary abnormalities in the extremely large *DMD* gene [11], which results in the almost complete loss of the Dp427‐M muscle isoform of the membrane‐associated cytoskeletal protein dystrophin [12].

This article is concerned with the proteomic analysis of dystrophinopathy [13] with special reference to membrane repair proteins. The article focuses on the mass spectrometry (MS) based proteomic analysis of annexin (ANX) proteins, notably ANXA isoforms [14], and the exploration of their role in myofiber repair, muscle regeneration and X‐linked muscular dystrophy. This includes the proteomic profiling of skeletal muscle‐associated ANXA proteins [15, 16, 17, 18], incorporating the description of membrane repair protein ANXA2 and related components [19, 20, 21, 22], as well as an outline of their crucial role in myofiber regeneration [14, 23, 24, 25].

## 2 Proteomic Identification and Characterization of Annexins

### 2.1 Classification of Annexin Isoforms

**FIGURE 1:**
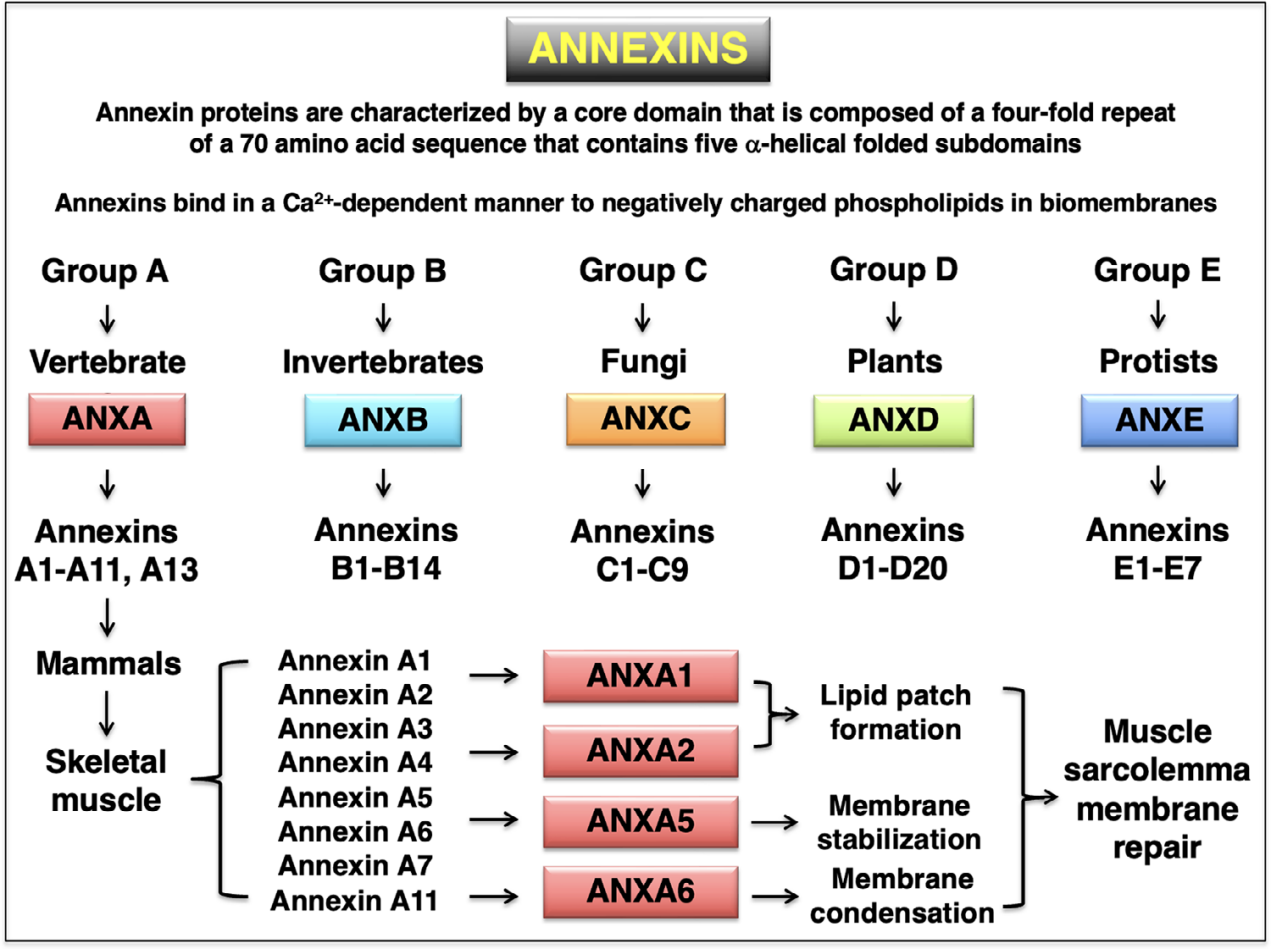
Overview of the annexin (ANX) superfamily of proteins that is characterized by their Ca^2+^‐dependent binding pattern to negatively charged phospholipids in biomembranes. Special focus is on the ANXA isoforms that are present in skeletal muscles.

Members of the ANX family of proteins are characterized by a core domain that is composed of a four‐fold repeat of an approximately 70 amino acid sequence that contains five alpha‐helical folded subdomain bundles [14]. A webpage with an extensive ANX sequence database and international projects, is available online (http://www.structuralchemistry.org/annexins/index.php). ANX proteins are defined and categorized based on their biochemical ability to specifically interact in a Ca^2+^‐dependent manner [26] with negatively charged phospholipid‐containing biomembranes [27]. Hence, the structurally related ANX isoforms share a canonical membrane binding mode consisting of three elements, that is, Ca^2+^‐ions, membrane domains and ANX proteins [28]. As summarized in Figure 1, ANX proteins can be divided into groups A (ANX A1‐A11, A13), B (ANX B1‐B14), C (ANX C1‐C9), D (ANX D1‐D20), and E (ANX E1‐E7) in vertebrates, invertebrates, fungi, plants and protists, respectively [14], plus ANX‐like proteins with remote sequence similarity in a small number of bacterial species [29].

The various ANX isoforms are involved in diverse biological functions related to cellular signaling cascades and membrane dynamics. ANX proteins were shown to play intricate roles in endocytosis, exocytosis, exosome formation, cell proliferation, interactions at the membrane‐cytoskeleton interface, mRNA transportation, translation and membrane repair [14, 26, 28]. The determination of the specific biochemical functions and the cellular localization of individual ANXA isoforms has established them as multi‐functional and multi‐compartment components with shared binding partners and related membrane binding mechanisms. The analysis of ANXA knockout mouse models [30] has revealed a certain degree of redundancy within the ANXA family of proteins [31]. The sensing capabilities of Ca^2+^, pH and lipid second messengers by certain ANXA isoforms probably form both a coordination hub for key cellular processes [15, 28, 32] and provide the first response to counteract detrimental plasma membrane damage [16, 17, 18].

**FIGURE 2:**
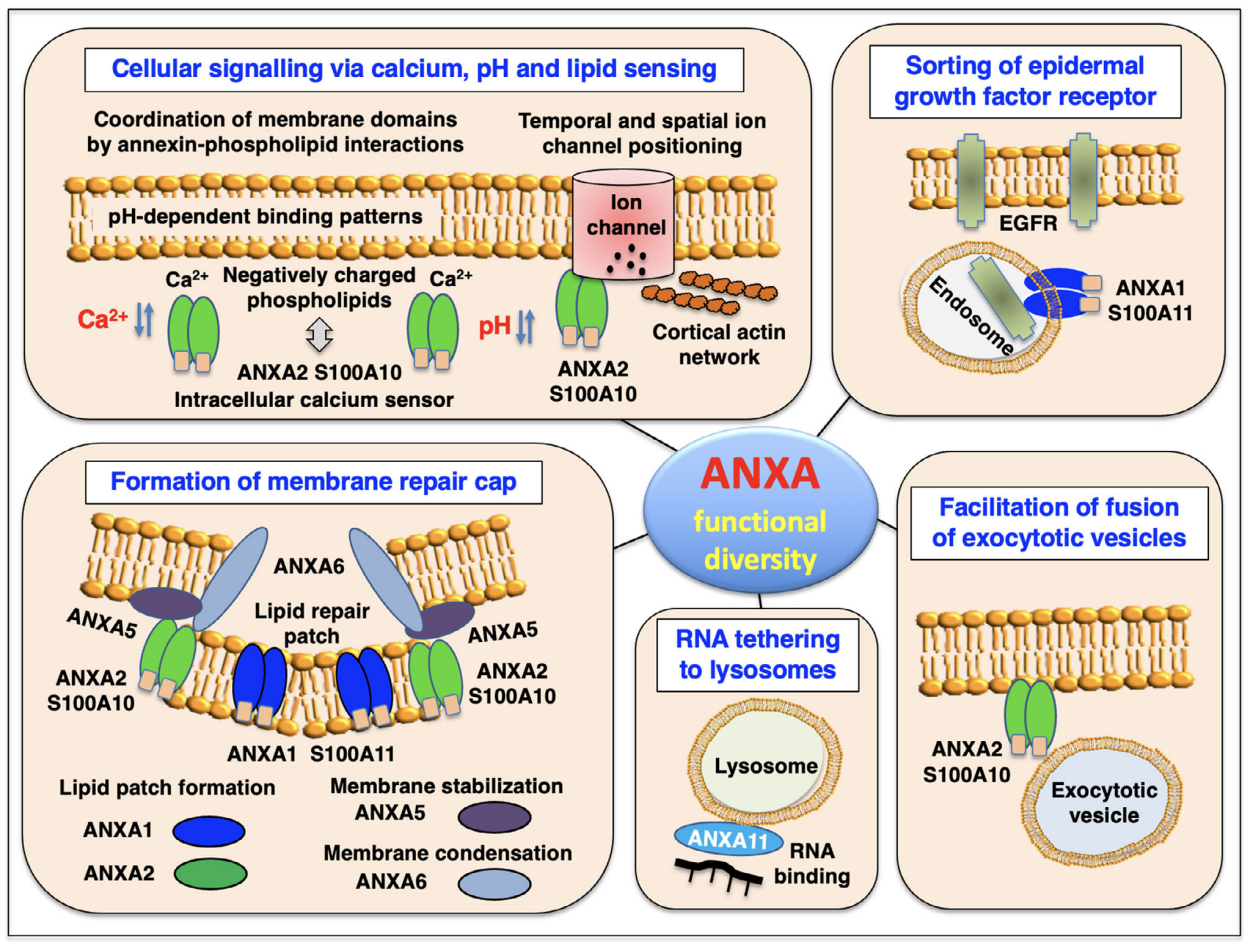
Overview of diverse capabilities and functions of annexins, including the sensing of Ca^2+^, pH and lipids and an integral role in coordinating crucial cellular processes and providing the first response to counteract detrimental plasma membrane damage.

There is a close relationship between elevated cytosolic Ca^2+^‐levels, altered pH‐values, changes in the three‐dimensional configuration of ANXA molecules and membrane docking within specific domains of the plasmalemma. Dynamic interactions between Ca^2+^‐regulated ANXA complexes and negatively charged phospholipids mediate the organization of distinct membrane domains, and are also involved in cellular membrane transport processes. Thus, ANXA units can be considered as Ca^2+^‐regulated membrane binding modules that facilitate the efficient sensing and subsequent fast response to cellular damage. Importantly, Ca^2+^‐ions serve as a physiological bridge between the lipid‐binding ANXA protein and acidic phospholipids, such as phosphatidic acid, phosphatidylserine and phosphatidylinositol 4,5‐bisphosphate. As summarized in Figure 2, the functional diversity of ANXAs ranges from calcium, pH and lipid sensing to sorting and positioning of ion channels and receptors, as well as the facilitation of fusion of exocytotic vesicles, RNA tethering to lysosomes and the formation of membrane repair cap structures following plasmalemmal rupturing [28]. ANXA2 appears to represent the most Ca^2+^‐sensitive member of this class of phospholipid‐binding proteins, and complex formation with S100A10 increases its biochemical availability for Ca^2+^‐induced translocation processes. Overall, the ANXA complement in skeletal muscles probably forms a complex network of highly sensitive Ca^2+^‐sensing units that regulate and influence key cellular signaling pathways [14, 15, 16, 17, 18].

### 2.2 Proteomic Identification of Annexin Isoforms in Skeletal Muscle

**FIGURE 3:**
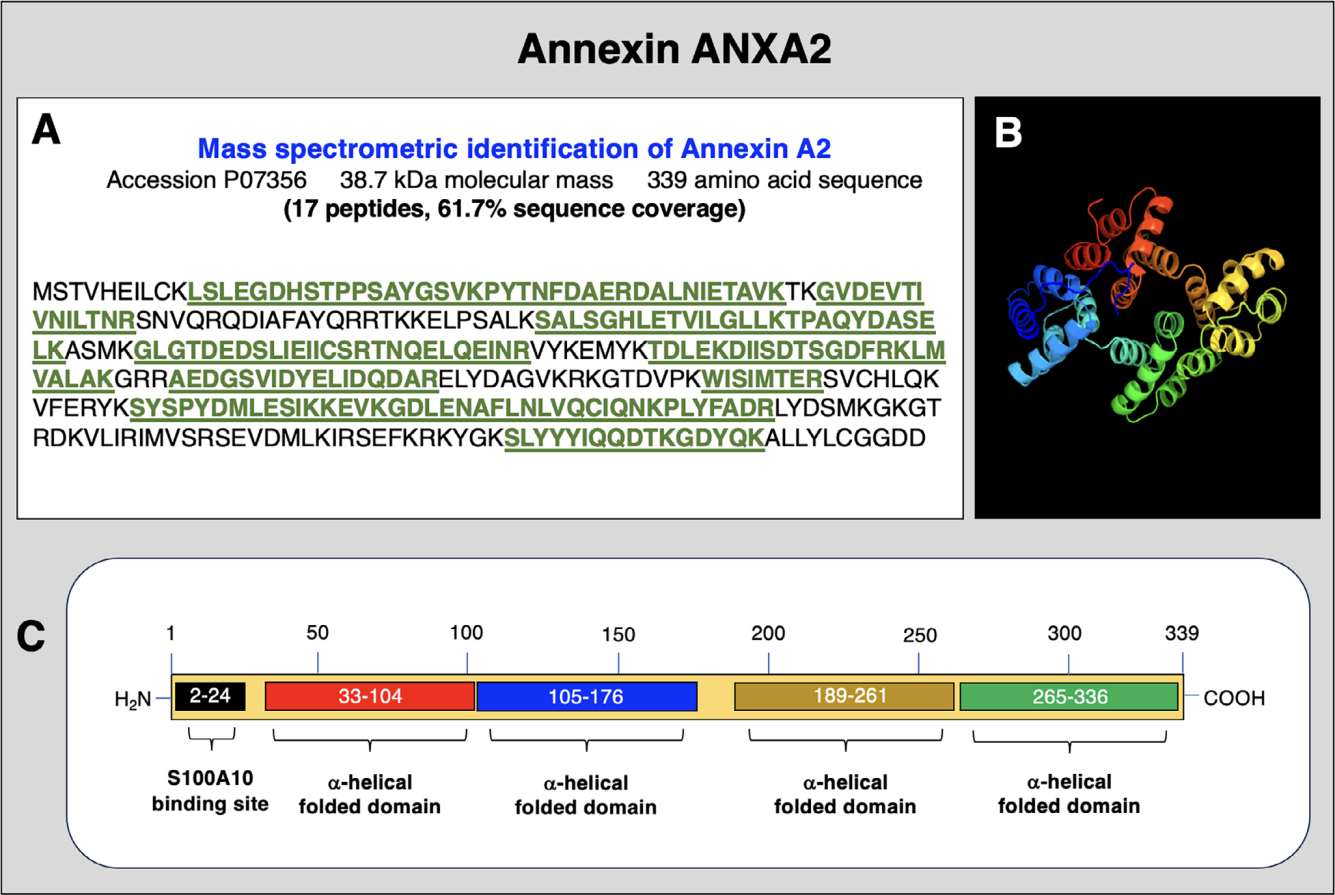
Presentation of the mass spectrometric identification of the ANXA2 isoform from skeletal muscle. Shown are (A) the amino acid sequence of ANXA2 and peptide sequences that were recognized by mass spectrometric analysis, (B) the proposed three‐dimensional molecular structure of ANXA2, and (C) the basic ANXA structure consisting of an amino‐terminal S100A1 binding site and four α‐helical folded domains. Proteomic analyses were carried out by routine methodology [41], and the image of the molecular structure of this ANXA isoform is based on Phyre2 analysis [43].

In skeletal muscle, 8 of the 12 highly conserved vertebrate ANXA isoforms (plus their splice variants) [14], have been identified by large‐scale proteomic cataloguing studies, namely ANXA1 to ANXA7 and ANXA11 [33, 34, 35], as listed in Figure 1. The novel insights into skeletal muscle biology that have been generated by proteomics and the various bioanalytical workflows that are routinely used in bottom‐up versus top‐down proteomic applications to study muscle‐associated proteins have been extensively reviewed [36, 37, 38]. The MS‐based identification of ANXA isoforms has included the systematic analysis of various voluntary muscles from humans (v*astus lateralis, trapezius* and *deltoideus*) and different animal species (*gastrocnemius, soleus, tibialis anterior, extensor digitorum longus*, diaphragm and extraocular muscles) [39, 40, 41, 42]. Figure 3 summarizes the MS‐based proteomic identification of ANXA2 isoform from murine skeletal muscle [41] and its proposed three‐dimensional molecular structure based on Phyre2 analysis [43].

Of note, various subtypes of skeletal muscles with differing fiber type compositions, including single muscle fibers, were investigated using proteomics and clearly identified a large number of ANXA isoforms [44, 45, 46, 47]. In humans, adult skeletal muscles can generally be categorized in up to three main types of contractile units with distinct kinetics and bioenergetics, that is, slow oxidative (type I), fast oxidative‐glycolytic (type IIa) and fast glycolytic (type IIx) [48]. This classification scheme correlates with the expression of specific myosin heavy chain (MyHC) isoforms: MyHC‐1, MyHC‐2a, and MyHC‐2x, which are encoded by the *MYH7*, *MYH2*, and *MYH1* genes, respectively [49]. Interestingly, ANXA5, unlike ANXA2 and ANXA6, was shown to be of higher abundance in human fast‐twitching glycolytic IIx myofibers, and found in association with dysferlin (DYSF) and dystrophin‐associated proteins [47].

Table 1 lists the MS‐based proteomic profiling of ANXA isoforms in various murine tissues and biological fluids. The MS data are based on optimized proteomic surveys [37] and the table lists the accession number and molecular mass of identified ANXA isoforms, plus percent sequence coverage and number of recognized peptides. ANXA1 to ANXA7 and A11 isoforms are present in the *gastrocnemius* muscle, diaphragm and the heart, while isoform ANXA3 appears to be absent from the extraocular muscle. Tongue muscles express ANXA1, ANXA2 and ANXA4 to ANXA6. For comparison, ANXA expression patterns in non‐muscle tissue extracts are shown, including brain, liver and spleen, as well as representative biofluids in the form of serum and urine. Interestingly, the ANXA2 isoform has been identified in all listed tissues and biological fluids (Table 1). Cellular localization studies have shown that ANXA proteins are associated with membranes, the cytosol and extracellular domains in skeletal muscles [50, 51, 52].

**TABLE 1:** Proteomic identification of annexin ANXA isoforms in mouse tissues and biofluids.

Accession number	Annexin isoform	Coverage %	Peptides	Molecular mass (kDa)
**Gastrocnemius muscle**				
P10107	ANXA1	39	11	38.7
P07356	ANXA2	62	17	38.7
O35639	ANXA3	54	14	36.4
P97429	ANXA4	49	11	35.9
P48036	ANXA5	50	14	35.7
P14824	ANXA6	70	46	75.8
Q07076	ANXA7	25	9	49.9
P97384	ANXA11	39	17	54.0
**Diaphragm muscle**				
P10107	ANXA1	39	9	38.7
P07356	ANXA2	50	15	38.7
O35639	ANXA3	37	11	36.4
P97429	ANXA4	37	9	35.9
P48036	ANXA5	61	16	35.7
P14824	ANXA6	61	36	75.8
Q07076	ANXA7	17	7	49.9
P97384	ANXA11	27	12	54.0
**Extraocular muscle**				
P10107	ANXA1	19	5	38.7
P07356	ANXA2	16	7	38.7
P97429	ANXA4	14	4	35.9
P48036	ANXA5	29	8	35.7
P14824	ANXA6	14	8	75.8
Q07076	ANXA7	2	1	49.9
P97384	ANXA11	8	3	54.0
**Tongue muscle**				
P10107	ANXA1	12	3	38.7
P07355	ANXA2	12	3	38.6
P97429	ANXA4	3	1	35.9
P48036	ANXA5	19	4	35.7
P14824	ANXA6	6	4	75.8
**Heart**				
P10107	ANXA1	4	1	38.7
P07356	ANXA2	63	20	38.7
O35639	ANXA3	55	13	36.4
P97429	ANXA4	31	7	35.9
P48036	ANXA5	49	12	35.7
P14824	ANXA6	59	32	75.8
Q07076	ANXA7	25	9	49.9
P97384	ANXA11	23	10	54.0
**Brain**				
P07356	ANXA2	29	8	38.7
O35639	ANXA3	19	6	36.4
P97429	ANXA4	8	2	35.9
P48036	ANXA5	58	13	35.7
P14824	ANXA6	57	29	75.8
Q07076	ANXA7	25	11	49.9
P97384	ANXA11	13	16	54.0
**Liver**				
P10107	ANXA1	12	3	38.7
P07356	ANXA2	37	7	38.7
O35639	ANXA3	9	1	36.4
P97429	ANXA4	49	15	35.9
P48036	ANXA5	67	19	35.7
P14824	ANXA6	66	38	75.8
Q07076	ANXA7	18	7	49.9
P97384	ANXA11	6	3	54.0
**Spleen**				
P10107	ANXA1	53	17	38.7
P07356	ANXA2	68	25	38.7
O35639	ANXA3	50	14	36.4
P97429	ANXA4	43	13	35.9
P48036	ANXA5	64	20	35.7
P14824	ANXA6	65	42	75.8
Q07076	ANXA7	31	14	49.9
P97384	ANXA11	35	16	54.0
**Serum**				
P07356	ANXA2	13	3	38.7
P48036	ANXA5	4	1	35.7
P14824	ANXA6	7	5	75.8
**Urine**				
P10107	ANXA1	8	2	38.7
P07356	ANXA2	18	5	38.7
P48036	ANXA5	24	6	35.7
P97384	ANXA11	28	13	54.0

*notes*: The mass spectrometric data are based on recent proteomic surveys of various tissues and biological fluids employing optimized proteomic approaches. Proteomic analyses were carried out by routine methodology [41].

## 3 Annexins and Muscle Membrane Repair

### 3.1 Protective Mechanisms to Counteract Muscular Damage

**FIGURE 4:**
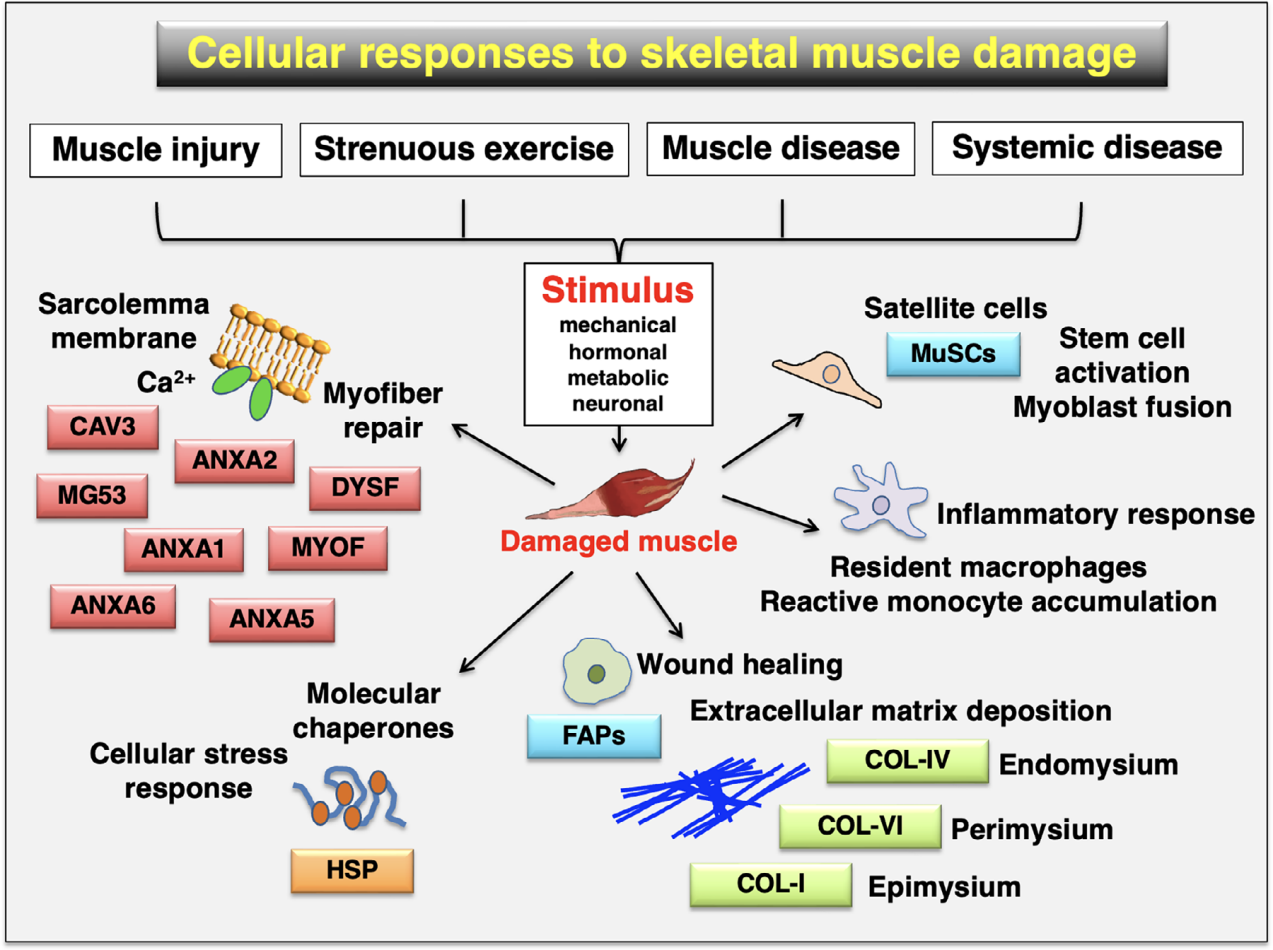
Overview of the diverse mechanisms that are activated in damaged skeletal muscles in response to myofiber injury, including (i) the Ca^2+^‐dependent and annexin‐associated sarcolemmal membrane repair system, (ii) the cellular stress response involving molecular chaperones such as heat shock proteins, (iii) the cellular wound healing system in conjunction with matrisomal remodeling and activation of fibro‐adipogenic progenitors within the extracellular matrix, (iv) the inflammatory response that is mediated by resident macrophages and infiltrating monocytes, and (v) the muscle stem cell population positioned between the sarcolemma and basal lamina that facilitates myofiber regeneration via satellite cell activation, proliferation and differentiation followed by myoblast fusion. ANXA, annexin A; CAV3, caveolin‐3; COL, collagen; DYSF, dysferlin; FAPs, fibro‐adipogenic progenitors; HSP, heat shock protein; MG53, Mitsugumin 53; MuSCs, muscle stem cells; MYOF, myoferlin.

Several inherent protective mechanisms exist to counteract continuous physical strain and skeletal muscle damage, which may occur alone or in combination, and are activated by mechanical, metabolic, hormonal, immunological and/or neuronal stimuli [53], as diagrammatically shown in Figure 4. These mechanisms include (i) the Ca^2+^‐dependent sarcolemmal myofiber repair machinery that recruits DYSF‐containing vesicles and forms membrane repair caps with the help of a variety of ANXA isoforms [54], (ii) the cellular stress response via molecular chaperoning systems, which involves diverse classes of heat shock proteins (HSPs), such as the HSP10s (HSPE), the small HSPs of 12–43 kDa (HSPB), the HSP60s (HSPD), the HSP70s (HSPA) and the large HSP90s (HSPC) [55], (iii) the intrinsic and reactive inflammatory response to myonecrotic changes mediated by resident macrophages and infiltration by monocytes and other immune cells in combination with the release of a variety of cytokines [56], (iv) muscle stem cell (MuSCs) mediated tissue regeneration involving satellite cell activation, proliferation and differentiation followed by fusion of myoblasts into myofibers [57], and (v) cellular wound healing mechanisms involving matrisomal remodeling and activation of fibro‐adipogenic progenitors (FAPs) [58] within the extracellular skeletal muscle cell niche, which is characterized by high levels of laminin in the basal lamina, collagen type IV (COL‐IV) in the endomysium, collagen type VI (COL‐VI) in the perimysium and collagen type I (COL‐I) in the epimysium [59].

Following injury, skeletal muscle regeneration initially involves the recruitment of macrophages during the inflammatory phase for the efficient removal of cellular debris and the release of pro‐inflammatory cytokines [56]. This is followed by the activation of crucial regenerative cell types in the skeletal muscle niche, including MuSCs, FAPs and endothelial cells [57, 58, 59, 60]. The differentiation of myogenic progenitors, myoblast fusion, FAPs differentiation and the release of growth factors from anti‐inflammatory and pro‐regenerative macrophages are then responsible for the resolution of the inflammatory phase [56]. The proliferation of endothelial cells, the recruitment of pericytes and the release of anti‐inflammatory cytokines are closely linked to the restorative phase of skeletal muscle regeneration. Finally, re‐vascularization and remodeling of the extracellular matrix play a crucial role in the establishment of a repaired myofiber environment [53].

### 3.2 Role of Annexin Isoforms A1, A2, A5, and A6 in Muscle Membrane Repair

A particular class of proteins that is found to be drastically increased during muscle membrane repair mechanisms [61], as well as being involved in various neuromuscular disorders [62], are the different isoforms of ANXA [14]. Ca^2+^‐dependent mechanisms are of central importance for repairing microlesions in the damaged sarcolemma membrane system, which may be associated with traumatic skeletal muscle injury, strenuous physical activities, primary neuromuscular disease or systemic diseases that affect the musculature in a secondary fashion [26]. Besides isoforms ANXA1, ANXA2, ANXA5 and ANXA6 [22, 23, 24, 25], other crucial muscle proteins that are involved in this process include DYSF [21, 63], a 237 kDa integral membrane protein of the ferlin family [64], caveolin‐3 (CAV3), the main structural component of caveolae domains in the sarcolemma [65], Mitsugumin 53 (MG53/TRIM72), a tripartite motif‐containing protein [66] and calpain‐3 (CAPN3), an intracellular and Ca^2+^‐dependent cysteine protease [67]. Within the large calpain (CAPN) family of proteases, calpain‐1 (CAPN1) and calpain‐2 (CAPN2) are found in most human cell types, while CAPN3 is expressed specifically in skeletal muscles.

CAPN1 and CAPN2 are implicated to be involved in membrane repair mechanisms by supporting the remodeling of the cytoskeletal network, particularly at the level of talin and vimentin, and additionally by contributing to the reduction in surface tension at the damaged sarcolemma [62]. CAPN3, which is also intrinsically involved in maintaining sarcomere integrity, plays a key role in the regulation of the DYSF‐containing complex during membrane repair [67]. Other components that were shown to contribute to DYSF complex formation include the 700 kDa structural scaffold protein desmoyokin, which is sensitive to the proteolytic activity of CAPN3 [68], the kinase‐binding protein affixin/β‐parvin which links integrin to the cytoskeleton [69] and myoferlin, a membrane‐associated Ca^2+^‐binding protein that belongs to the ferlin family with regulatory functions in membrane fusion [64, 70]. The above listed scaffolding protein desmoyokin is encoded by the *AHNAK* gene, and thus also referred to as AHNAK protein, whereby “ahnak” means “giant” in Hebrew.

**FIGURE 5:**
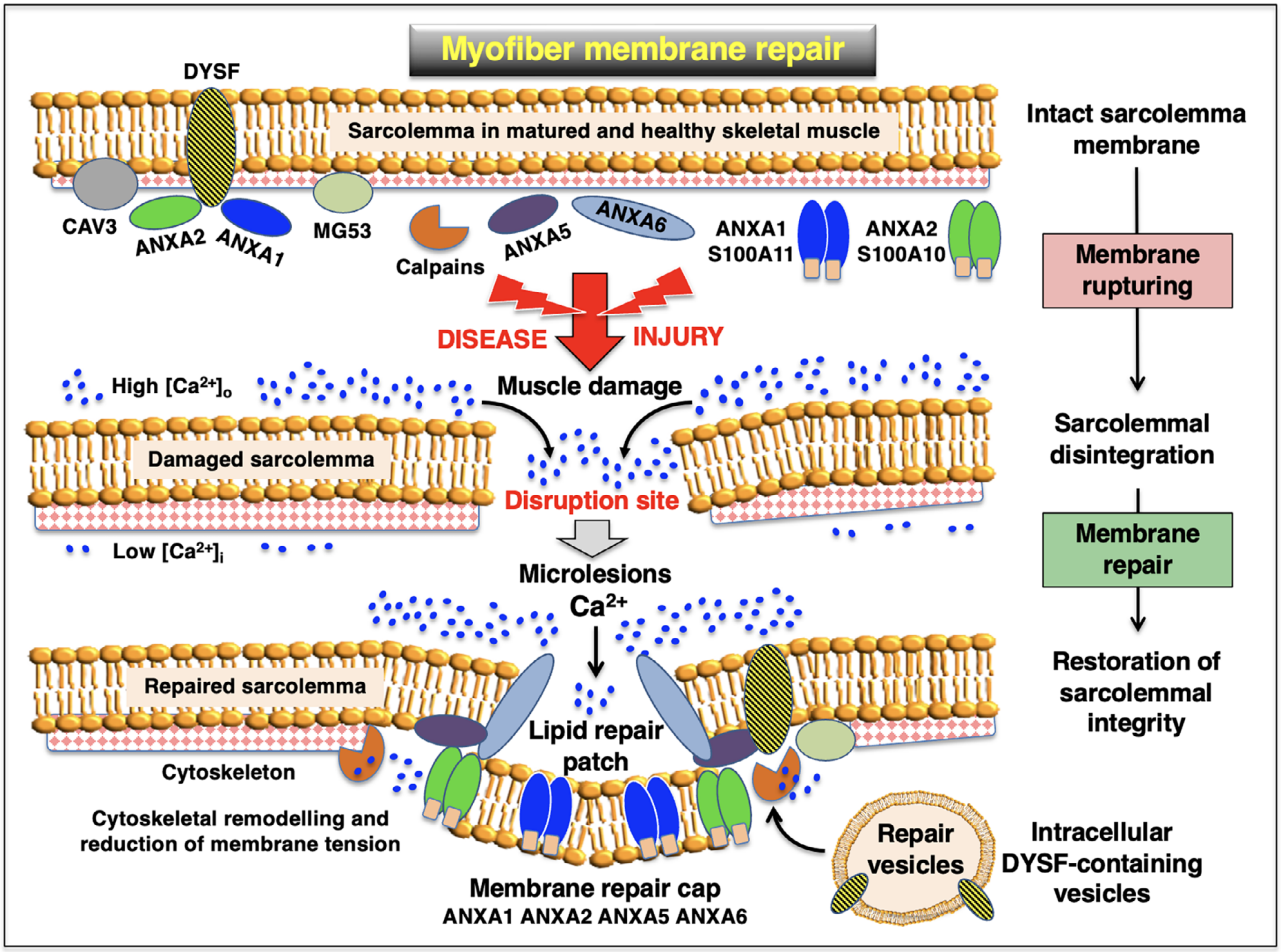
Schematic overview of the Ca^2+^‐dependent sarcolemmal myofiber repair process that is based on the swift recruitment of intracellular and DYSF‐containing vesicles and the formation of membrane repair caps that encompass a variety of ANXA isoforms. Shown is the sealing of detrimental membrane gaps due to microlesions via fusion of lipid patches. Tissue damage triggers aggregation of repair vesicles and the production of lipid patches that fuse at the disruption site with the leaky plasma membrane. During the patch repair response, the proteins MG53 and ANXA5 are recruited at the edges of the ruptured zone, and vesicular aggregation occurs via the recruitment of ANXA1 and ANXA2. The local activation of specific calpain enzymes modulates DYSF action and triggers cytoskeletal remodeling and altered sarcolemmal tension. The membrane repair cap contains ANXA isoforms A1, A2, A5, and A6. ANXA, annexin A; CAV3, caveolin‐3; DYSF, dysferlin; MG53, Mitsugumin 53.

Figure 5 provides an overview of the involvement of ANXA isoforms and associated repair proteins in the sealing of detrimental gaps due to microlesions. The fusion of lipid patches plays a central role in plugging leaky domains in skeletal muscle membranes. In adult and undamaged myofibers, DYSF localizes both to the sarcolemma, where it interacts with ANXA1 and ANXA2, which in turn may bind to CAV3, and to intracellular repair vesicles [71]. In addition, the sarcolemmal region contains ANXA5, ANXA6, ANXA1/S100A11 hetero‐tetramers and ANXA2/S100A10 hetero‐tetramers [19], as well as inactive proteases. Membrane‐associated DYSF is recruited in an actin cytoskeleton‐dependent way [61, 72]. Upon skeletal muscle damage, the DYSF‐containing vesicles undergo aggregation and produce lipid patches that fuse at the disruption site with the damaged sarcolemma. This is a Ca^2+^‐dependent process that is initiated by the massive influx of Ca^2+^‐ions through the leaky surface membrane [64].

The patch repair response occurs at elevated intracellular Ca^2+^‐levels leading to the oligomerization of MG53 [73] and consists of the deposition of MG53 and ANXA5 at the edges of the ruptured membrane [74], the induction of an sarcolemmal curvature to form a tight seal at the repair cap domain [17] and vesicular aggregation via the recruitment of ANXA1 and ANXA2 [21]. The increased Ca^2+^‐concentration also triggers the local activation of specific calpain isoforms, which results in the proteolytic cleavage of cytoskeletal elements and thereby reduces membrane tension at the disruption site [62] and also plays a role in the proteolytic cleavage of DYSF to generate a 72 kDa C‐terminal protein derivative called mini‐dysferlinC72 [75]. Thus, the formation of a membrane repair cap containing DYSF, MG53, ANXA1, ANXA2, ANXA5, and ANXA6, as well as CAV3, affixin and AHNAK, is crucial for myofiber membrane repair [65].

The ANXA repertoire that is routinely identified in skeletal muscle preparations by MS‐based analyses using both bottom‐up and top‐down proteomic approaches is listed in Table 2. The application of gel‐based [76] versus gel‐free [77] protein separation techniques in skeletal muscle proteomics, as well as the main MS‐based methodologies, have recently been reviewed in detail [37]. The isoforms ANXA1 to ANXA7 and ANXA11 have been identified in skeletal muscles by proteomics [33, 34, 39, 45], as also listed in the above Table 1. In addition, ANXA8 was detected using deep proteomic screening approaches that have identified over 10,000 muscle‐associated protein species [35]. Table 2 summarizes the findings from proteomic cataloguing studies using biopsy material, whole muscle homogenates, muscle cell lines and single myofiber preparations. This encompasses the proteomic profiling of human *vastus lateralis*, *trapezius* and *deltoideus* muscles and various biopsy samples [33, 34, 36, 40, 42, 78, 79], single human myofiber preparations [47, 80, 81, 82], rodent g*astrocnemius*, *soleus*, *tibialis anterior*, *triceps brachii*, *extensor digitorum longus* and diaphragm muscles [39, 41, 83, 84, 85, 86], the murine C2C12 muscle cell line [35], and single mouse myofiber preparations [44, 45, 46, 87].

**TABLE 2:** Mass spectrometry‐based proteomic identification of annexin ANXA isoforms in normal human and animal skeletal muscles.

Mass spectrometric analyses	Analytical approaches	Annexin identification	References
Proteomic cataloguing studies of human muscles (*vastus lateralis*, *trapezius*, *deltoideus*, and various biopsy samples)	1D‐GeLC, 2D‐GE, 2D‐DIGE, MALDI‐ToF MS, LC‐MS/MS	ANXA1, ANXA2, ANXA3, ANXA4, ANXA5, ANXA6, ANXA7, ANXA11	[33, 34, 40, 42, 78, 79]
Proteomic single human myofiber analysis of varying age	FASP, LC‐MS/MS	ANXA1, ANXA2, ANXA3, ANXA4, ANXA5, ANXA6, ANXA7, ANXA11	[47, 80, 81, 82]
Proteomic cataloguing studies of rodent muscles (g*astrocnemius*, *soleus*, *tibialis anterior*, *triceps brachii*, *extensor digitorum longus*, and diaphragm)	2D‐GE, 2D‐DIGE, SILAC, MALDI‐ToF MS, LC‐MS/MS	ANXA1, ANXA2, ANXA3, ANXA4, ANXA5, ANXA6, ANXA7, ANXA8, ANXA11	[35, 39, 41, 83, 84, 85, 86]
Deep proteomic profiling of murine C2C12 muscle cell line (myoblasts)	FASP, LC‐MS/MS	ANXA1, ANXA2, ANXA3, ANXA4, ANXA5, ANXA6, ANXA7, ANXA8, ANXA11	[35]
Proteomic single mouse myofiber analysis (*vastus lateralis*, *extensor digitorum longus*, and *soleus*)	LC‐MS/MS, FASP, 1‐step in‐solution digestion	ANXA1, ANXA2, ANXA3, ANXA4, ANXA5, ANXA6, ANXA7, ANXA11	[44, 45, 46, 87]

*Note*: Listed are major proteomic cataloguing studies that have identified annexin ANXA isoforms in normal skeletal muscle tissues.

Abbreviations: 1D‐GeLC, one‐dimensional gel electrophoresis liquid chromatography; 2D‐GE, two‐dimensional gel electrophoresis; ANXA, annexin A; DIGE, difference in‐gel electrophoresis; FASP, filter‐aided sample preparation; LC‐MS/MS, liquid chromatography tandem mass spectrometry; MALDI, matrix assisted laser desorption/ionization; ToF, Time‐of‐Flight.

## 4 Annexins and Duchenne Muscular Dystrophy

A variety of ANXA isoforms were identified in human and animal skeletal muscles that exhibit significantly altered expression levels in X‐linked muscular dystrophy, including isoforms ANXA1 to ANXA7 and ANXA11, as determined by comparative proteomics using mostly analytical bottom‐up approaches [41, 88, 89, 90, 91, 92, 93, 94, 95, 96, 97, 98, 99, 100, 101, 102, 103, 104]. Table 3 lists the main findings in relation to ANXA expression levels in dystrophin‐deficient muscle specimens from patients suffering from dystrophinopathy and various dystrophic animal models. In general, proteomic screening studies were carried out with biopsy specimens from DMD and Becker muscular dystrophy (BMD) patients [88, 89, 105, 106, 107, 108], as well as established animal models, including the *mdx‐23* mouse [109], the *mdx‐4cv* mouse [110], the GRMD dog [111], and the DMD pig [112].

**TABLE 3:** Comparative mass spectrometry‐based proteomic analysis of annexin ANXA isoforms in dystrophic skeletal muscles.

Mass spectrometric analyses	Analytical approaches	Annexin identification	References
Proteomic profiling of *vastus lateralis* biopsies from DMD/BMD patients	2D‐DIGE, MALDI‐ToF MS, LC‐MS/MS	Increased ANXA2, ANXA5 and ANXA11 in DMD muscle	[88]
Proteomic analysis of hind limb muscle (cytosol) from the *mdx‐23* mouse	2D‐GE, MALDI‐ToF MS	Increased ANXA5 in *mdx‐23* muscle	[90]
Proteomic profiling of *mdx‐23* mouse diaphragm extracts	2D‐DIGE, LC‐MS/MS	Increased ANXA5 in *mdx‐23* diaphragm	[91]
Proteomic profiling of *mdx‐4cv* mouse hind limb muscle extracts	LC‐MS/MS	Increased ANXA1, ANXA2, ANXA5, ANXA6 and ANAX7 in *mdx‐4cv* hind limb	[92]
Proteomic profiling of *mdx‐23* mouse *quadriceps femoris*	Density ultra‐centrifugation, SDS‐PAGE, on‐membrane digestion, LC‐MS/MS	Increased ANXA1 in *mdx‐23* muscle	[93]
Proteomic profiling of *mdx‐23* mouse diaphragm versus extraocular muscle	TMT isobaric mass tagging, MudPIT, LC‐MS/MS	Increased ANXA1 and ANXA5 in *mdx‐23* diaphragm	[94]
Proteomic profiling of *vastus lateralis* from dystrophic GRMD dog model	ICAT, LC‐MS/MS	Increased ANXA1 and ANXA2 in GRMD dog muscle	[95]
Proteomic survey of *biceps femoris* from dystrophic GRMD dog model following allogenic MuStem cell application	SDS‐PAGE, ICPL, LC‐MS/MS	Increased ANXA1 and ANXA5 due to MuStem cell induction	[96]
Proteomic profiling of *mdx‐23* and *mdx‐52* mouse *tibialis anterior*	TMT isobaric mass tagging, LC‐MS/MS	Increased ANXA1 and ANXA4 in dystrophic mouse models	[97]
Proteomic profiling of the aged *mdx‐4cv* mouse diaphragm	FASP, LC‐MS/MS	Increased levels of ANXA1, ANXA2, ANXA3, ANXA4, ANXA5 and ANXA7 in *mdx‐4cv* diaphragm	[98]
Proteomic analysis of skeletal muscles (*triceps brachii*) from the DMD pig model	LC‐MS/MS	Expression changes in ANXA1, ANXA2, ANXA4, ANXA5, ANXA6, ANXA7 and ANXA11 in DMD pig	[99]
Comparative proteomic survey of the *mdx‐23* mouse diaphragm, *soleus*, *extensor digitorum longus*, *flexor digitorum brevis*, and *interosseus*	LC‐MS/MS	Increased ANXA2 and ANXA5 in *mdx‐23* muscles	[100]
Proteomic profiling of *mdx‐4cv* mouse *gastrocnemius* and C2C12 myoblasts	LC‐MS/MS	Increased ANXA1 and ANXA5 in dystrophin‐deficient muscle cells	[101]
Proteomic analysis of *gastrocnemius* muscle from the *mdx‐23* mouse model	SILAM mouse analysis, LC‐MS/MS	Increased ANXA2, ANXA5 and ANXA6 in *mdx‐23* mouse muscle	[102]
Proteomic analysis of *gastrocnemius* muscle from the *mdx‐23* and *mdx‐52* mouse models	SILAM mouse analysis, LC‐MS/MS	Increased ANXA1 and ANXA2 in dystrophic mouse muscle	[103]
Proteomic analysis of the sarcolemma‐enriched fraction from dystrophic *mdx‐4cv* skeletal muscle	Differential centrifugation, lectin agglutination, LC‐MS/MS	Increased ANXA1 in the dystrophin‐deficient *mdx‐4cv* sarcolemma	[104]

*Note*: Listed are major proteomic studies that have clearly identified significant changes in annexin ANXA isoforms in dystrophic muscle tissues.

Abbreviations: 2D‐GE, two‐dimensional gel electrophoresis; ANXA, annexin A; DIGE, difference in‐gel electrophoresis; DMD/BMD, Duchenne/Becker muscular dystrophy; FASP, filter‐aided sample preparation; ICAT, isotope‐coded affinity tag; ICPL, isotope‐coded protein labeling; LC‐MS/MS, liquid chromatography tandem mass spectrometry; MALDI, matrix assisted laser desorption/ionization; MudPIT, multi‐dimensional protein identification technology; MuStem, allogeneic muscle‐derived stem cells; SDS‐PAGE, sodium dodecyl sulfate polyacrylamide gel electrophoresis; SILAM, stable isotope labeling in mammals; TMT, tandem mass tag; ToF, Time‐of‐Flight.

### 4.1 Duchenne Muscular Dystrophy

A large number of inherited neuromuscular disorders have been classified [6], including severe diseases with distinct primary abnormalities in genes that encode abundant skeletal muscle proteins [8]. Membrane repair dysfunction has been shown to occur primarily in the limb‐girdle muscular dystrophies of *CAPN3*‐related LGMD‐1L (LGMD‐D4) and LGMD‐2A (LGMD‐R1), *DYSF*‐related LGMD‐2B (LGMD‐R2), *ANO5*‐related LGMD‐2L (LGMD‐R12) and *CAV3*‐related LGMD‐1C (rippling muscle disease) [113]. The involvement of abnormal expression patterns of CAV3, DYSF, CAPN3 and anoctamin‐5, in conjunction with aberrant ANXA isoform levels, in these muscular disorders has been recently reviewed in detail by Croissant et al. [62]. Interestingly, the proteomic analysis of dysferlinopathy revealed increases in ANXA1, ANXA2, ANXA4 and ANXA5 in patient biopsy specimens, possibly reflecting a compensatory process due to deficiency in DYSF [114].

In contrast to the above listed muscular dystrophies with mutated genes that encode membrane repair proteins, highly progressive DMD is due to genetic abnormalities in the *DMD* gene [11, 115], causing the almost complete loss of the full‐length dystrophin protein isoform Dp427‐M in voluntary muscles [9]. The absence of dystrophin is related to membrane fragility and sarcolemmal micro‐rupturing. The more benign form of dystrophinopathy, BMD, is associated with variable abundance and/or size of cytoskeletal dystrophin and is characterized by a later onset of muscle wasting symptoms. X‐linked muscular dystrophy can be considered a primary skeletal muscle disease, but also exhibits body‐wide abnormalities affecting the functioning of the heart, respiratory system, skeleton, gastro‐intestinal tract, kidneys and liver, as well as the peripheral and central nervous system [116]. The potential of MS‐based proteomics to determine the highly complex pathophysiological crosstalk in X‐linked muscular dystrophy and its associated multi‐system dysfunction has been critically examined in a recent review [117].

The cellular pathogenesis of skeletal muscle degeneration in DMD is characterized with progressive myofiber necrosis [118] that is triggered by the loss of dystrophin in the membrane cytoskeleton and accompanying collapse of the dystrophin‐associated glycoprotein complex [119], consisting of dystroglycans, sarcoglycans, sarcospan, dystrobrevins, and syntrophins, as well as laminin‐211 and actin [13]. Since the dystrophin‐glycoprotein complex, in conjunction with the integrin complex of the sarcolemma, provides a stabilizing linkage between the cortical actin cytoskeleton and the basal lamina component laminin in normal muscle fibers [12], the deficiency in dystrophin triggers membrane rupturing during the ongoing strains of excitation‐contraction‐relaxation cycles. Myofiber degeneration is followed by a chronic inflammatory phenotype [120], which in turn causes reactive myofibrosis in the form of high levels of collagen deposition and associated components of the extracellular matrix [121]. Of note, specific myofibrotic signatures provoke distinct effects on myoblast activity [122]. The loss of dystrophin appears to severely affect MuSCs integrity, instigating satellite cell dysfunction and thereby weakening the regenerative capacity of dystrophic skeletal muscles [117, 123].

### 4.2 Annexin Expression in Dystrophin‐deficient Skeletal Muscle

Proteomic studies have identified a certain degree of apparently compensatory increases in both cytoskeletal proteins, such as vimentin, desmin and tubulins [41], and molecular chaperones, especially the category of small HSPs (HSPB), such as cardiovascular cvHSP (HSPB7) and alphaB‐crystallin (HSPB5) [13]. This could be interpreted as an up‐regulation of protective mechanisms by the dystrophic muscle to strengthen the dystrophin‐deficient cytoskeletal network by increasing intermediate filaments and microtubules, as well as rescue or remove misfolded proteins by the action of the molecular chaperoning system [118]. It is therefore not surprising that key members of the membrane repair machinery, such as ANXAs and DYSF, are increased in dystrophic myofiber populations [41, 88, 90, 91, 92, 93, 94, 95, 96, 97, 98, 99, 100, 101, 102, 103, 104]. This section focuses on a select number of proteomic investigations of dystrophin‐deficient muscles that have identified altered expression levels of ANXA isoforms (Table 3). Comprehensive listings of altered proteins other than ANXAs from major proteomic analyses of dystrophic skeletal muscles have previously been published [124, 125, 126].

The systematic proteomic profiling of dystrophin‐deficient human skeletal muscle specimens has identified a large number of protein alterations in dystrophinopathy [88, 89, 105, 106, 107, 108]. Recently, a comparative proteomic study of DMD/BMD patient specimens by da Silva et al. [108] has clearly established the expression levels of ANXA1, ANXA2, ANXA5, ANXA6, ANXA7, and ANXA11 in human skeletal muscles and demonstrated an interesting concomitant decrease in sarcomeric proteins and increase in ubiquitination‐related components in dystrophinopathy, as well as alterations of proteins involved in energy metabolism, cellular respiration and RNA processing. The comparative proteomic profiling of *vastus lateralis* biopsies from DMD patients revealed increased ANXA2, ANXA5 and ANXA11 levels in severely dystrophic skeletal muscles [88]. Overall, the analysis of patient samples using both two‐dimensional difference in‐gel electrophoresis (2D‐DIGE) and liquid chromatography tandem MS (LC‐MS/MS) established a large number of alterations in proteins belonging to the cytoskeleton, sarcomere, extracellular matrix, glycolytic pathway, mitochondrial bioenergetics, lipid metabolism and protein homeostasis [88]. This underlines the enormous complexity of proteome‐wide changes [13] that are triggered by the loss of the cytoskeletal protein dystrophin [10].

One of the earliest applications of proteomics in the field of dystrophic animal model research focused on the MS‐based analysis of hind limb muscle extracts [90] from the spontaneous *mdx‐23* mouse that harbors a point mutation in exon 23 of the murine *Dmd* gene [109]. The study identified an increase in ANXA5, besides characteristic elevations in HSPs and creatine kinase, and a drastic reduction in adenylate kinase isoform AK1 [90]. This was confirmed by the proteomic profiling of the dystrophic and highly fibrotic *mdx‐23* mouse diaphragm muscle employing fluorescence 2D‐DIGE and LC‐MS/MS analysis [91]. The analysis of *mdx‐23 quadriceps femoris* muscle using a combination of differential centrifugation, density ultra‐centrifugation, sodium dodecyl sulphate polyacrylamide gel electrophoresis (SDS‐PAGE) and on‐membrane digestion showed a clear increase in ANXA1 in dystrophic mouse muscle [93]. A comparative survey of the severely dystrophic *mdx‐23* diaphragm versus only mildly affected *mdx‐23* extraocular muscles, which employed tandem mass tag (TMT) isobaric mass tagging, multi‐dimensional protein identification technology (MudPIT) and LC‐MS/MS, revealed increased ANXA1 and ANXA5 levels in the *mdx‐23* diaphragm [94]. The proteomic profiling of *mdx‐23* versus *mdx‐52* mouse *tibialis anterior* muscle specimens revealed increased ANXA1 and ANXA4 levels in dystrophic mouse models [97]. Proteomic analyses of *gastrocnemius* muscle from the *mdx‐23* mouse model using the stable isotope labeling in mammals (SILAM) method revealed increased levels of isoforms ANXA2, ANXA5 and ANXA6 [102] and ANXA1 and ANXA2 [103] in dystrophic skeletal muscles. The parallel analysis of several different subtypes of skeletal muscles from the *mdx‐23* mouse, that is, diaphragm, *soleus*, *extensor digitorum longus*, *flexor digitorum brevis*, and *interosseus*, identified increases in ANXA2 and ANXA5 in dystrophic muscles [100]. Besides ANXA2, this comparative study of mildly versus severely affected *mdx‐23* muscles identified lamin and vimentin as universal dystrophic markers that are independent of the degree of disease progression.

Proteomic profiling of hind limb muscle extracts from another *mdx*‐type murine model of dystrophinopathy, the *mdx‐4cv* mouse, demonstrated elevated ANXA1, ANXA2, ANXA5, ANXA6, and ANXA7 levels in dystrophic muscles [92]. The *mdx‐4cv* mouse has been generated by a chemically induced nonsense point mutation in exon 53, which causes a premature termination of translation [110]. The skeletal muscles from *mdx‐4cv* mice exhibit considerably less revertant myofibers as compared to the *mdx‐23* mouse. Studies with *mdx‐4cv* mouse *gastrocnemius* muscle and murine C2C12 myoblasts also revealed increased ANXA1 and ANXA5 levels in dystrophin‐deficient muscle cells [101]. The MS‐based analysis of young versus senescent *mdx‐4cv* mouse diaphragm confirmed drastically increased levels of isoforms ANXA1 to ANXA5 and ANXA7 at all ages [98]. The subproteomic analysis of the sarcolemma‐enriched fraction from dystrophic *mdx‐4cv* skeletal muscle, using differential centrifugation, lectin affinity agglutination of highly enriched sarcolemma vesicles and LC‐MS/MS analysis, identified an increased ANXA1 concentration in the dystrophin‐deficient sarcolemma membrane [104].

Proteomic analysis of the *vastus lateralis* muscle from the dystrophic GRMD dog model of dystrophinopathy [111] utilized isotope‐coded affinity tag (ICAT) to show increased ANXA1 and ANXA2 levels due to dystrophin deficiency in golden retrievers [95]. A related proteomic survey of *biceps femoris* muscle from the dystrophic GRMD model, following allogenic muscle‐derived stem cell (MuStem) application with isotope‐coded protein labeling (ICPL), revealed additional increases in ANXA1 and ANXA5 due to MuStem cell induction [96]. The analysis of the *triceps brachii* muscle from the severe DMD pig model [112] identified expression changes in isoforms ANXA1, ANXA2, ANXA4 to ANXA7, and ANXA11 [99].

Regarding the presence of ANXA isoforms in biofluids, previous studies have established them in serum, saliva and urine. As listed in the above Table 1, ANXA2, ANXA5, and ANXA6 can be found in serum, and ANXA1, ANXA2, ANXA5, and ANXA11 are present in urine under normal physiological conditions. Of note, a recent study on the response to prednisone has established increased serum ANXA1 levels as a potential biomarker of intermittent steroid treatment in muscular dystrophy [127].

The above listed studies clearly establish ANXA as a promising biomarker candidate in dystrophic skeletal muscle tissues, where it plays an intrinsic role in initiating repair mechanisms to counteract dystrophin deficiency and the resulting loss of sarcolemmal integrity. This includes the lipid patch‐forming ANXA1 and ANXA2 isoforms, as well as ANXA5, that participates in the strengthening of the weakened sarcolemma and ANXA6, which mediates membrane condensation at the disruption site [14]. Verification studies using immunoblotting confirmed ANXA increases in muscular dystrophy, in conjunction with characteristic changes in fibrotic markers such as collagen COL‐VI and periostin [98]. Proteomic surveys of non‐muscle tissues have also revealed changes of ANXA, such as the kidney in the *mdx‐4cv* mouse model of DMD [128].

**FIGURE 6:**
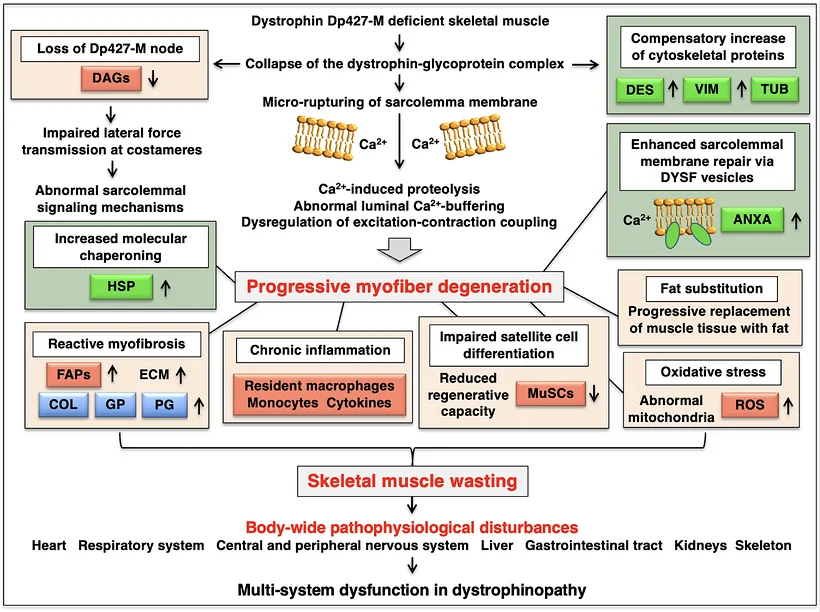
Schematic presentation of major steps involved in the molecular and cellular pathogenesis of Duchenne muscular dystrophy. Deficiency of dystrophin and its associated glycoprotein complex leads to micro‐rupturing of the sarcolemma and accompanying chronic influx of Ca^2+^‐ions into the sarcoplasm. This triggers Ca^2+^‐dependent proteolysis and renders myofibers more susceptible to necrosis. Chronic muscle degeneration is associated with impaired excitation‐contraction coupling, diminished satellite cell differentiation, inflammation, oxidative stress, fat substitution and reactive myofibrosis. Compensatory mechanisms that counteract the dystrophic phenotype include the increased expression of cytoskeletal proteins, an enhanced cellular stress response and up‐regulation of the membrane repair machinery involving DYSF‐containing vesicles and the ANXA‐mediated production of membrane repair caps. ANXA, annexin A; COL, collagen; DAGs, dystrophin‐associated glycoproteins; Dp427‐M, muscle dystrophin of 427 kDa; DES, desmin; DYSF, dysferlin; ECM, extracellular matrix; FAPs, fibro‐adipogenic progenitors; GP, glycoproteins; HSP, heat shock protein; MuSCs, muscle stem cells; MYOF, myoferlin; PG, proteoglycans; ROS, reactive oxygen species; TUB, tubulin; VIM, vimentin.

Crucial aspects of the molecular and cellular pathogenesis of X‐linked muscular dystrophy are summarized in Figure 6, which outlines the increase in ANXAs of the membrane repair machinery in the context of other adaptations and dysfunctions in dystrophic myofibers. The collapse of the dystrophin‐glycoprotein complex and associated micro‐rupturing of the weakened sarcolemma initiates a Ca^2+^‐dependent degradation process. Abnormal Ca^2+^‐cycling through the sarcoplasmic reticulum is based on impaired transverse tubular voltage sensing, Ca^2+^‐uptake, luminal Ca^2+^‐buffering, cytosolic Ca^2+^‐binding and triadic Ca^2+^‐release [129]. Progressive myofiber degradation is linked to (i) sterile inflammation with increased levels of macrophage activity, (ii) abnormal mitochondrial functioning and oxidative stress, (iii) a progressive replacement of muscle tissue with adipose tissue, (iv) reactive myofibrosis that is characterized by the increased deposition of collagens, proteoglycans and glycoproteins of the extracellular matrix, and (v) an impaired regenerative capacity of dystrophin‐lacking skeletal muscles due to abnormal satellite cell differentiation [10, 13, 98, 116, 118, 129, 130]. Natural protective mechanisms to compensate dystrophin deficiency include the up‐regulation of the molecular chaperoning system to counteract cellular stress and protein misfolding, the increase of cytoskeletal components to stabilize weakened myofibers, the partial substitution of dystrophin with the extra‐junctional expression of its autosomal homologue utrophin, and enhanced sarcolemmal membrane repair via DYSF‐containing vesicles and ANXA‐mediated lipid patching of the disruption site.

## 5 Conclusion

This review article has outlined the MS‐based proteomic profiling of DMD with a special focus on the ANXA family of proteins, which are intrinsically involved in plasmalemma repair and skeletal muscle regeneration. The biochemical, proteomic and cell biological analysis of the myofiber membrane repair system has established that it consists of a large set of diverse proteins, including ANXA1, ANXA2, ANXA5, and ANXA6, as well as CAV3, MG53, CAPNs, and DYSF. Following cellular injury or disease, microlesions at the sarcolemma are swiftly restored by a Ca^2+^‐driven process that involves the production of an ANXA‐containing membrane repair cap to counteract the detrimental effects of sustained skeletal muscle damage. The systematic MS‐based proteomic analysis of both muscle biopsy material from DMD patients and diverse skeletal muscle preparations from established animal models of dystrophinopathy has revealed increased levels of distinct ANXAs, including isoforms ANXA1 to ANXA5 and ANXA7, in dystrophic skeletal muscle tissues. Hence, muscular dystrophy‐associated alterations in ANXA expression levels form part of the comprehensive biomarker signature of skeletal muscle changes/adaptations due to deficiency in the membrane cytoskeletal protein dystrophin. Alterations in ANXA isoforms and associated membrane repair proteins might therefore be suitable to improve the differential diagnosis, prognosis and therapeutic monitoring of DMD. Since the up‐regulation of membrane repair proteins can be interpreted as an adaptive response in dystrophic muscles, therapy‐induced decreases in ANXAs, MG53, CAV‐3, and/or DYSF could potentially serve as a useful indicator for restored sarcolemmal integrity and reduced membrane micro‐rupturing.

## Author Contributions

All authors made a significant contribution to the work reported. Draft manuscript preparation was carried out by Paul Dowling and Kay Ohlendieck. All authors reviewed and edited the drafts and approved the final version of the manuscript.

## Conflicts of Interest

The authors declare no conflicts of interest.

## Data Availability

Data sharing is not applicable to this article, as no datasets were generated or analyzed during the current study.
